# Association between Intraoperative Early Warning Score and Mortality and In-Hospital Stay in Lower Gastrointestinal Spontaneous Perforation

**DOI:** 10.1155/2023/8910198

**Published:** 2023-08-29

**Authors:** Kazuya Takada, Yusuke Nagamine, Akira Ishii, Yan Shuo, Takumi Seike, Hanako Horikawa, Kentaro Matsumiya, Tetsuya Miyashita, Takahisa Goto

**Affiliations:** ^1^Department of Anesthesiology, Yokohama City University Medical Center, 4-57 Urafune-cho, Minami-ku, Yokohama 232-0024, Kanagawa, Japan; ^2^Department of Anesthesiology and Critical Care Medicine, Yokohama City University Hospital, 3-9 Fukuura, Kanazawa-ku, Yokohama 236-0004, Kanagawa, Japan

## Abstract

**Background:**

Early warning scores (EWSs) can be easily calculated from physiological indices; however, the extent to which intraoperative EWSs and the corresponding changes are associated with patient prognosis is unknown. In this study, we investigated whether EWS and the corresponding time-related changes are associated with patient outcomes during the anesthetic management of lower gastrointestinal perforation.

**Methods:**

This was a single-center, retrospective cohort study conducted at a tertiary emergency care center. Adult patients who underwent surgery for spontaneous lower gastrointestinal perforations between September 1, 2012, and December 31, 2019, were included. The National Early Warning Score (NEWS) and Modified Early Warning Score (MEWS) were calculated based on the intraoperative physiological indices, and the associations with in-hospital death and length of hospital stay were investigated.

**Results:**

A total of 101 patients were analyzed. The median age was 70 years, and there were 11 cases of in-hospital death (mortality rate: 10.9%). There was a significant association between the intraoperative maximum NEWS and in-hospital death (odds ratio (OR): 1.60, 95% confidence interval (CI): 1.10–2.32, *p*=0.013) and change from initial to maximum NEWS (OR: 1.60, 95% CI: 1.07–2.40, *p*=0.023) in the crude analysis. However, when adjustments were made for confounding factors, no statistically significant associations were found. Other intraoperative EWS values and changes were not significantly associated with the investigated outcomes. The preoperative sepsis-related organ failure assessment score and the intraoperative base excess value were significantly associated with in-hospital death.

**Conclusions:**

No clear association was observed between EWSs and corresponding changes and in-hospital death in cases of lower gastrointestinal perforation. The preoperative sepsis-related organ failure assessment score and intraoperative base excess value were significantly associated with in-hospital death.

## 1. Introduction

Lower gastrointestinal perforation peritonitis is an acute condition frequently encountered in the emergency surgery field. It is a complex condition with several underlying causes and different prognoses. The incidence of multiple organ failure syndrome (MOFS) in this patient population has been reported to be up to 73%, with a mortality rate of 30% [1, 2], and although significant advances in medical technology have been made over the past few years, the mortality rate in cases of perforation peritonitis remains high [3, 4].

Predicting the prognosis of perforation peritonitis is clinically useful for optimizing strategies, such as surgery or admission to the intensive care unit (ICU), for appropriate treatment. Several risk factors, such as age over 70 years, preoperative hypotension, and perioperative blood transfusions, have been reported to be associated with mortality in cases of perforation peritonitis [5, 6]; however, most of these studies were focused on preoperative and postoperative factors, and very few studies have examined the association between intraoperative factors and prognosis in colorectal perforation. On the other hand, several studies have reported that abnormal intraoperative physiological parameters, such as tachycardia, hypotension, hypertension, and hypothermia, are associated with perioperative complications and mortality following elective surgery [7, 8]; however, the association between the intraoperative physiological indices and prognosis of critically ill patients who undergo emergency surgery is not clear. Moreover, single physiological indices are not considered sufficient to predict an increased risk in the emergency field; therefore, attempts have been made to accurately predict the prognosis by scoring and weighting various physiological indices [9].

Morgan et al. developed the Early Warning Score (EWS), a physiological score that can be measured at the bedside, to enable the detection of deteriorating conditions and the appropriate utilization of medical resources in the early stages of acute illnesses, including those occurring in and out of the hospital [10]. The Modified Early Warning Score (MEWS) was developed subsequently to exclude urinary volume and other factors for easier measurement [9]. The National Early Warning Score (NEWS) was developed by bringing together all 33 of these variations [11, 12]; its usefulness has been validated in the emergency department and in predicting the worsening of medical and surgical inpatient conditions [13, 14], leading to its widespread adoption [15]. It has recently been recognized as a useful tool in screening for sepsis [16], and the use of EWS has been mentioned in the surviving sepsis campaign guidelines [17]. EWS has also been studied for its time variability. Levin et al. reported that multiple EWS measurements can predict the mortality of patients admitted to the emergency department more accurately than individual measurements [18]. However, although it has been widely validated, it is unclear whether the EWS calculated based on intraoperative vital signs and its time course is associated with the prognosis in patients with spontaneous lower gastrointestinal perforations.

Thus, the aim of this study was to determine (1) the intraoperative physiological indices and their weighted scoring, represented by the EWS, during anesthetic management of lower gastrointestinal perforation and (2) whether the corresponding time-related changes are associated with patient outcomes.

## 2. Materials and Methods

### 2.1. Study Design, Settings, and Patients

This was a single-center, retrospective cohort study conducted at a tertiary emergency medical care center. Adult patients who underwent surgery for spontaneous lower gastrointestinal perforations between September 1, 2012, and December 31, 2019, were included. Perforation of the lower gastrointestinal tract was defined as tract perforation beyond the ligamentum Treitz. Patients diagnosed with upper gastrointestinal perforation based on the intraoperative findings, patients with an unknown perforation site, trauma cases, and acute postoperative cardiovascular surgery cases were excluded.

### 2.2. Exposure

The intraoperative vital signs and intraoperative laboratory data were extracted from the electronic anesthesia charts for the calculation of MEWS/NEWS. During each event, the vital signs at every hour after the start of surgery were extracted, and NEWS and MEWS were calculated based on the corresponding values.  S1 Table in the Supplementary Materials shows the calculation of NEWS. MEWS was calculated according to Utah MEWS as reported previously (S2 Table) [18].

The start-to-end delta MEWS and NEWS were calculated by subtracting the MEWS and NEWS values at the start of surgery from those at the end of surgery. They ranged from 8 (clinically worsening trend) to −8 (clinically improving trend). The start-to-max delta MEWS and NEWS were defined as the maximum values minus the NEWS and MEWS at the start of surgery. They ranged from 8 (clinically worse) to 0 (clinically unchanged).

The preoperative sepsis-related organ failure assessment (SOFA) score [19] was calculated using the preoperative examination results and vital signs before entering the operating room, while the partial pressure of oxygen (PaO_2_) was estimated using oxygen saturation (SpO_2_) values according to a conversion table (S3 Table), as it was not measured preoperatively in some cases. The intraoperative parameter data (lactate levels and base excess (BE)) were obtained from the electronic anesthesia charts, with the first measured value after entering the operating room defined as the start value, and the value just before the end of surgery defined as the end value. The maximum and minimum values during surgery were defined as the max and min values.

### 2.3. Outcomes

The primary outcome was in-hospital death. The secondary outcome was the length of hospital stay. Cases of in-hospital deaths were excluded from the analysis of the length of hospital stay.

### 2.4. Statistical Analysis

Multivariate logistic regression analysis was used to examine the association between the physiological measures and in-hospital death and the length of hospital stay. We adjusted for age, sex, preoperative comorbidity (Charlson comorbidity index (CCI)) [20], and the SOFA score as potential confounding factors in the statistical analysis. We did not perform a sample size analysis as the maximum number of cases that could be collected during the time period was included in this study. Statistical significance was set at a *p* value of less than 0.05. Missing values were not imputed. All statistical procedures were performed using STATA version 17.0 SE-Standard Edition (Texas, USA).

### 2.5. Ethical Consideration

This study was approved by the Institutional Ethics Review Board of Yokohama City University (approval no. B201100037; chairperson: Dr. Shin Maeda; approval date: December 8, 2020). The requirement of informed consent was waived by the ethics committee (Yokohama City University). The opportunity to withdraw consent was provided on the website. All procedures were carried out in accordance with the Declaration of Helsinki.

## 3. Results

**Figure 1:**
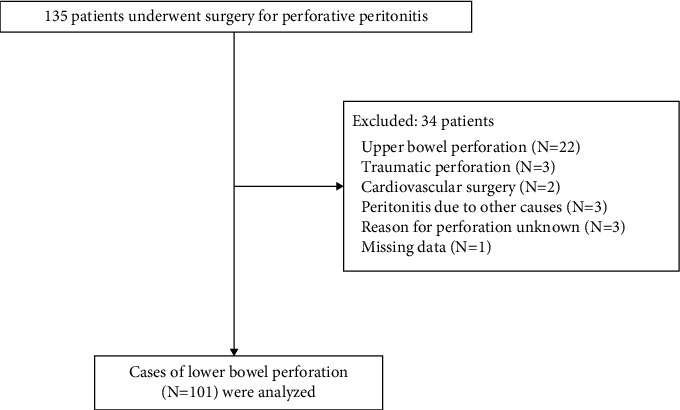
Study enrollment flowchart.

A total of 135 cases were initially included, of which 34 met the exclusion criteria; therefore, 101 cases were finally analyzed (Figure 1).  Table 1 summarizes the patient characteristics. The median age was 70 years, and 53.5% of the patients were males. The median preoperative SOFA score was 2 (interquartile range (IQR): 1–5), and there were 11 in-hospital deaths (mortality rate: 10.9%). The median length of hospital stay in cases without in-hospital deaths was 28 days (IQR: 14–55).

### 3.1. MEWS and NEWS

Table 2 presents the MEWS and NEWS data.  Table 3 presents the results of multivariate logistic regression analysis for in-hospital death and multivariate linear regression analysis for the length of hospital stay, respectively. The max NEWS (crude odds ratio (OR): 1.60, 95% confidence interval (CI): 1.10–2.32, *p*=0.013) and start-to-max delta NEWS, representing the initial-to-maximum change in NEWS (crude OR: 1.60, 95% CI: 1.07–2.40, *p*=0.023) were significantly associated with in-hospital death. However, no statistically significant associations were found when the analysis was adjusted for confounding factors. No statistically significant associations with in-hospital death or length of hospital stay were found for the other MEWS and NEWS values (Table 3).

### 3.2. Preoperative SOFA, Intraoperative BE, and Lactate Level

The preoperative SOFA score was found to be significantly associated with in-hospital death and the length of hospital stay. After adjusting for confounders (age, sex, and CCI), the adjusted OR for in-hospital death was 1.18 (95% CI: 1.00–1.35, *p*=0.046) and the adjusted multivariate linear regression coefficient for the length of hospital stay was 5.60 (95% CI: 2.86–8.34, *p* < 0.05) (Table 4).

BE at the start of surgery (adjusted OR: 0.80, 95% CI: 0.67–0.96, *p*=0.014), the minimum intraoperative BE (adjusted OR: 0.69, 95% CI: 0.54–0.89, *p*=0.004), and the start-to-end difference in BE (adjusted OR: 1.30, 95% CI: 1.01–1.68, *p*=0.038) were found to be significantly associated with in-hospital death; there was no significant association with the length of hospital stay. Lactate level was associated with in-hospital death only at initiation (adjusted OR: 1.29, 95% CI: 1.03–1.61, *p*=0.029).

No statistically significant association was found between pulse rate, blood pressure, or temperature and outcome at the start of the procedure, at the end of the procedure, or at the change (S4 Table).

## 4. Discussion

In this study, we tested our hypothesis that intraoperative worsening of physiological indices could be associated with a poorer prognosis in cases of acute lower gastrointestinal perforation. Contrary to our hypothesis, we found no association between the absolute values and the trends of the physiological parameters and the weighted physiological scores, that is, the NEWS and MEWS and in-hospital death or the length of hospital stay. However, we observed that the preoperative SOFA score, intraoperative BE, and lactate level at the beginning of the surgery were associated with in-hospital death.

The maximum NEWS and start-to-max delta NEWS showed statistically significant associations with in-hospital death in the crude analysis; however, these were not found to be statistically significant with the adjusted multivariate analysis. Among the confounding factors used for adjustment, the preoperative SOFA score has been reported to be associated with prognosis in patients with intestinal perforation, and similar results were obtained in the present study [21]. We considered the possibility that the maximum NEWS and the preoperative SOFA score were strongly associated, as adjustment for the preoperative SOFA score resulted in the association between the maximum NEWS and outcome no longer remaining statistically significant. Univariate linear regression analysis with the maximum NEWS as the outcome and the preoperative SOFA score as the explanatory variable yielded a beta coefficient of 0.11 (95% CI: 0.01–0.21, *p* = 0.027), suggesting an association. These results suggest that even in emergency cases wherein organ failure cannot be adequately assessed using the SOFA score, abnormal intraoperative NEWS (high maximum NEWS or worsening NEWS) may help predict the prognosis from the intraoperative stage.

No association with the outcome was observed for NEWS, MEWS, and the physiological indices or their temporal changes after adjustment for the SOFA score. A previous study reported that the temporal changes in pulse pressure, respiratory rate, and systolic blood pressure were associated with in-hospital mortality in the emergency department [22], and for EWS, temporal worsening has been reported to be associated with prognosis [18]. These discrepancies may at least in part be accounted for the effects of surgery and general anesthesia on EWS. Our patients were mechanically ventilated and paralyzed during general anesthesia intraoperatively, which likely compromised the prognostic value of EWS as the respiratory rate was reported to have the highest prognostic accuracy among the vital signs of early warning scores [23]. The appearance and progression of acidosis in the present study were also strongly associated with mortality. The failure to assess the respiratory rate, which may indirectly reflect the acid-base equilibrium, may have significantly impacted the results.

Body temperature is also affected by anesthesia. General anesthetics and opioids affect autonomic thermoregulation and lower body temperature [24]. Opioids and volatile anesthetics decrease the febrile response by suppressing inflammatory cytokines [25, 26]. It is suggested that this underlying mechanism may have modified the temperature abnormality in gastrointestinal perforation, resulting in overestimation in the hypothermic region and underestimation in the hyperthermic region.

Consciousness was not evaluated during the surgery as the patient was under sedation. A previous study reported that in ViEWS, the predecessor of NEWS, the short version score, which excludes the level of consciousness, and the full score are equivalent in their ability to predict consciousness [27]. Regardless, the inability to assess the level of awareness may not yield a significant influence. It is possible that these measures were not related to the outcomes as they could not accurately reflect the severity of illness and are attributable to modification by continuous sedation and controlled breathing.

We were unable to demonstrate prognostic relevance for EWS and each of the physiological indices. One possible reason why physiological indices and their changes were not found to be associated with prognosis in this study could be the hemodynamic changes due to anesthetic management. In emergency cases, the anesthesiologist often constantly adjusts the infusion volume and catecholamine and the anesthetic dosage during surgery, which dynamically influences the hemodynamic status of the patient. Therefore, it may be difficult to predict the prognosis from temporal changes in vital signs alone in severe cases. It is not possible to determine whether maintaining the intraoperative physiological indices improves the prognosis, and our study results do not suggest that anesthesia management that improves intraoperative vital signs should be neglected.

Intraoperative BE and its change and the lactate level at the start of surgery were found to be associated with in-hospital death. Lawton et al. observed the time course of BE measured intraoperatively and postoperatively in patients undergoing scheduled major surgery and reported a trend toward longer ICU stays with worsening BE [28]. Since their study included ICU patients who had undergone major surgeries, the number of severe cases was low. For the severe emergency cases included in this study, we were able to show an association with in-hospital death, as most causes of metabolic acidosis are presumed to be associated with anaerobic metabolism or renal dysfunction. Our results also showed that, even during the short intraoperative period, the temporal changes in BE were associated with the prognosis. Previous reports have shown an association between preoperative BE and mortality in patients with gastrointestinal perforation [29]; however, no studies have shown an association between intraoperative BE over time and prognosis. Thus, this is the first study to show an association between the time course of intraoperative BE and prognosis in patients with lower gastrointestinal tract perforation. Serum lactate has been studied for its prognostic and early diagnostic utility in patients with severe sepsis, and it has been reported to have a prognostic value for patients with colorectal perforation [30], similar to the present results. However, there was no significant difference in the lactate level at the end of surgery or its time course, which is contrary to the results of previous studies. In addition to the base lactate value, a previous study reported that the 24-hour lactate clearance was associated with the best prognosis [30], suggesting that changes over a longer period of time, rather than changes during the short intraoperative period, may reflect prognosis. The present results suggest that BE may be more sensitive than the lactate level as an intraoperative prognostic predictor for critically ill patients. Quantitative analysis of the components of acidosis in septic patients indicates that the earlier metabolic acidosis was mainly due to a strong ion gap resulting from unmeasured ions [31]. The elevation of nonvolatile acids in septic patients may result primarily from renal or hepatic dysfunction. Our results suggest that during the early phase of the pathogenesis of lower gastrointestinal perforation, metabolic acidosis followed by elevated nonvolatile acidosis may be more apparent earlier than the elevated lactate due to tissue hypoperfusion; however, this could not be demonstrated in the present study.

This is the first study to examine the physiological indices in critically ill patients during surgery, their temporal changes, and their association with prognosis; thus, this study provides valuable data highlighting the association between the changes in BE during surgery and prognosis. Nevertheless, there are several limitations to our study. This was a single-center study conducted at a tertiary care hospital, and the patient condition may have been worse than that in other studies. Furthermore, as the number of cases was limited, more accurate results may be obtained by increasing the sample size and including more facilities in the future.

## 5. Conclusions

In cases of lower gastrointestinal perforation and the maximum and initial-to-maximum change in the NEWS, a weighted physiological parameter score was significantly associated with in-hospital death in unadjusted analysis but not after adjustment for confounding factors. We observed that the preoperative SOFA score, intraoperative BE, and lactate level at the beginning of the surgery were associated with in-hospital death.

## Figures and Tables

**Table 1:** Baseline characteristics of the patients.

	Total (*N* = 101)	Missing
Age (years)^a^	70 [61–77]	0
Male^b^	54 (53.5)	0
BMI^a^	21.1 [18.8–23.3]	0
ASA 1–2^b^	43 (42.5)	0
ASA > 3^b^	58 (57.5)	0
Charlson comorbidity index^a^	2 [1–3]	0
Preoperative SOFA score^a^	2 [1–5]	0
Anesthesia time (min)^a^	215 [174–262]	0
Operation time (min)^a^	143 [106–192]	0
Bleeding (ml)^a^	100 [0–350]	0
In-out balance (ml)^a^	2625 [1705–3540]	0
Use of catecholamine, intraoperative^b^	49 (48.5)	0
Open surgery^b^	78 (77.2)	0
Laparoscopic surgery^b^	23 (22.8)	0
Cancer^b^	31 (30.7)	0
Length of ICU stay (days)^a^	4 [1–9]	0
ICU admission^b^	78 (77.2)	0

^a^Continuous variables, presented as median (interquartile range). ^b^Categorical variables, presented as number (%). BMI, body mass index; ASA, American Society of Anesthesiologists; SOFA, sequential organ failure assessment; ICU, intensive care unit.

**Table 2:** Intraoperative MEWS, NEWS, base excess values, and lactate levels.

	Total	Missing
*MEWS*		
Start MEWS, median	3 [1–4]	9
Max MEWS, median	3 [3–4]	5
End MEWS, median	2 [1–3]	7
Start-to-end delta MEWS, median	0 [−1–0]	10
Start-to-1-hour delta MEWS, median	−1 [−1 to 1]	10
Start-to-max delta MEWS, median	1 [0–2]	9

*NEWS*		
Start NEWS, median	8.5 [8–9.5]	9
Max NEWS, median	10 [9–11]	5
End NEWS, median	8 [7–10]	7
Start-to-end delta NEWS, median	0 [−1 to 1]	10
Start-to-1-hour delta NEWS, median	0 [−1 to 1]	10
Start-to-max delta NEWS, median	1 [0–2]	9

*Base excess*		
Start base excess, median	−2.2 [−5 to −0.2]	18
End base excess, median	−3.9 [−5.6 to −1.7]	18
Minimum base excess, median	−4.2 [−6.6 to −2.2]	18
Start-to-end delta base excess, median	−1.2 [−2.6 to 0.8]	18

*Lactate levels*		
Start lactate, median	2.2 [1–3]	18
End lactate, median	2.2 [1–3]	19
Max lactate, median	2.5 [1.4–4.2]	18
Start-to-end delta lactate, median	0.1 [−0.3 to 0.6]	19

All variables are continuous variables presented as median with interquartile range.

**Table 3:** Multivariate logistic regression analysis of NEWS and MEWS for in-hospital death and the length of hospital stay.

	In-hospital death		Length of hospital stay	
	Crude OR (95% CI), *p* value	Adjusted^a^ OR (95% CI), *p* value	Crude linear regression coefficient (95% CI), *p* value	Adjusted^a^ linear regression coefficient (95% CI), *P* value
*MEWS*				
Start MEWS	1.03 (0.65–1.64), *p*=0.870	1.00 (0.57–1.73), *p*=0.999	−0.57 (−7.51–6.37), *p*=0.870	−0.19 (−6.91–6.55), *p*=0.956
Max MEWS	1.37 (0.91–2.05), *p*=0.128	1.14 (0.73–1.78), *p*=0.565	1.30 (−5.50–8.11), *p*=0.705	−0.69 (−7.45–6.05), *p*=0.838
End MEWS	1.20 (0.81–1.77), *p*=0.368	1.049 (0.69–1.57), *p*=0.817	−0.35 (−7.49–6.78), *p*=0.922	−0.78 (−7.72–6.17), *p*=0.824
Start-to-end delta MEWS	1.15 (0.79–1.70), *p*=0.460	1.042 (0.70–1.55), *p*=0.835	0.23 (−6.42–6.89), *p*=0.945	−0.73 (−7.45–5.98), *p*=0.828
Start-to-1-hour delta MEWS	1.23 (0.80–1.88), *p*=0.344	0.989 (0.57–1.69), *p*=0.968	3.15 (−3.25–9.56), *p*=0.330	1.28 (−5.16–7.72), *p*=0.694
Start-to-max delta MEWS	1.44 (0.93–2.24), *p*=0.104	1.19 (0.72–1.97), *p*=0.486	1.51 (−7.23–10.25), *p*=0.731	−1.51 (−10.48–7.47), *p*=0.739

*NEWS*				
Start NEWS	1.21 (0.79–1.85), *p*=0.371	1.14 (0.71–1.82), *p*=0.595	−1.13 (−7.51–5.26), *p*=0.726	−1.62 (−7.74–4.49), *p*=0.599
Max NEWS	1.60 (1.10–2.32), *p*=0.013	1.41 (0.94–2.12), *p*=0.093	1.66 (−4.04–7.36), *p*=0.565	−0.72 (−6.47–5.04), *p*=0.805
End NEWS	1.31 (0.94–1.83), *p*=0.111	1.25 (0.87–1.79), *p*=0.224	−0.65 (−6.60–5.31), *p*=0.830	−1.12 (−6.97–4.73), *p*=0.704
Start-to-end delta NEWS	1.16 (0.83–1.63), *p*=0.378	1.14 (0.81–1.61), *p*=0.451	1.78 (−4.50–8.06), *p*=0.575	0.31 (−5.54–6.16), *p*=0.918
Start-to-1-hour delta NEWS	1.28 (0.85–1.91), *p*=0.230	1.19 (0.76–1.86), *p*=0.442	2.03 (−4.40–8.46), *p*=0.531	1.52 (−4.78–7.84), *p*=0.631
Start-to-max delta NEWS	1.60 (1.07–2.40), *p*=0.023	1.49 (0.92–2.42), *p*=0.107	1.77 (−5.83–9.37), *p*=0.644	0.00 (−7.76–7.77), *p*=0.999

^a^Adjusted for age, sex, preoperative sepsis-related organ failure assessment score, and Charlson comorbidity index. OR, odds ratio; 95% CI, 95% confidence interval.

**Table 4:** Multivariate logistic regression analysis of the preoperative SOFA score and intraoperative BE and the lactate level for in-hospital death and the length of hospital stay.

	In-hospital death		Length of hospital stay	
	Crude OR (95% CI), *p* value	Adjusted OR (95% CI), *p* value	Crude linear regression coefficient (95% CI), *p* value	Adjusted linear regression coefficient (95% CI), *p* value
Preoperative SOFA score^b^	1.17 (1.03–1.34), *p*=0.018	1.18 (1.00–1.35), *p*=0.046	5.61 (3.04–8.18), *p* < 0.01	5.60 (2.86–8.34), *p* < 0.01
Start BE^a^	0.85 (0.75–0.96), *p*=0.012	0.80 (0.67–0.96), *p*=0.014	−0.77 (−3.53–1.99), *p*=0.582	0.85 (−1.95–3.65), *p*=0.545
End BE^a^	0.85 (0.71–1.02), *p*=0.073	0.83 (0.68–1.02), *p*=0.070	−0.24 (−3.55–3.07), *p*=0.885	0.93 (−2.28–4.16), *p*=0.564
Minimum BE^a^	0.77 (0.66–0.92), *p*=0.003	0.69 (0.54–0.89), *p*=0.004	−1.03 (−4.36–2.29), *p*=0.537	0.48 (−2.8–3.79), *p*=0.771
Start-to-end delta BE^a^	1.26 (1.02–1.56), *p*=0.031	1.30 (1.01–1.68), *p*=0.038	2.35 (−3.11–7.81), *p*=0.393	0.55 (−6.01–4.90), *p*=0.841
Start lactate^a^	1.25 (1.03–1.53), *p*=0.024	1.29 (1.03–1.61), *p*=0.029	5.44 (−0.22 to −11.10), *p*=0.059	3.18 (−2.57–8.95), *p*=0.273
End lactate^a^	1.04 (0.95–1.14), *p*=0.377	1.03 (0.93–1.15), *p*=0.540	2.42 (0.12–4.71), *p*=0.039	1.41 (−0.89–3.71), *p*=0.226
Max lactate^a^	1.00 (0.95–1.06), *p*=0.981	0.99 (0.94–1.05), *p*=0.920	1.19 (0.19–2.11), *p*=0.019	0.72 (−0.25–1.69), *p*=0.145
Start-to-end delta lactate^a^	0.89 (0.60–1.30), *p*=0.538	0.85 (0.52–1.39), *p*=0.528	2.33 (−0.56–5.22), *p*=0.112	−1.29 (−1.54–4.12), *p*=0.365

^a^Adjusted for age, sex, preoperative SOFA score, and Charlson comorbidity index. ^b^Adjusted for age, sex, and Charlson comorbidity index. SOFA, sepsis-related organ failure assessment; OR, odds ratio; 95% CI, 95% confidence interval; BE, base excess.

## Data Availability

The datasets used to support the findings of this study are available from the corresponding author upon reasonable request.

## Supplementary Materials

Supplementary Materials S1 Table: National Early Warning Score (NEWS) calculation. S2 Table: Utah Modified Early Warning Score (MEWS) calculation. S3 Table: oxygen saturation (SpO_2_) to partial pressure of oxygen (PaO_2_) conversion. S4 Table: vital signs and outcome adjusted for age, sex, preoperative SOFA score, and Charlson comorbidity index. STROBE Statement—Checklist of items that should be included in reports of cohort studies.
