# Supplementary material for: Association between Intraoperative Early Warning Score and Mortality and In-Hospital Stay in Lower Gastrointestinal Spontaneous Perforation
Source: Anesthesiol Res Pract. 2023 Aug 29;2023:8910198. doi: 10.1155/2023/8910198 (PMC10480023; doi:10.1155/2023/8910198)
Supplement: Supplementary Materials — S1 Table: National Early Warning Score (NEWS) calculation. S2 Table: Utah Modified Early Warning Score (MEWS) calculation. S3 Table: oxygen saturation (SpO2) to partial pressure of oxygen (PaO2) conversion. S4 Table: vital signs and outcome adjusted for age, sex, preoperative SOFA score, and Charlson comorbidity index. STROBE Statement—Checklist of items that should be included in reports of cohort studies. [file 8910198.f1.zip › S1_Table_file.docx]

S1 Table. National Early Warning Score (NEWS) calculation

| Score | 3 | 2 | 1 | 0 | 1 | 2 | 3 |
| --- | --- | --- | --- | --- | --- | --- | --- |
| Respiration rate, /min | ≤8 |  | 9–11 | 12–20 |  | 21–24 | ≥25 |
| SpO_2_ Scale 1, (%) | ≤91 | 92–93 | 94–95 | ≥96 |  |  |  |
| SpO_2_ Scale 2, (%) | ≤83 | 84–85 | 86–87 | 88–92  ≥93 on Air | 93–94  on Oxygen | 95–96  on Oxygen | ≥97  on Oxygen |
| Air or oxygen? |  | Oxygen |  | Air |  |  |  |
| Systolic BP, mmHg | ≤90 | 91–100 | 101–110 | 111–219 |  |  | ≥220 |
| Pulse, /min | ≤40 |  | 41–50 | 51–90 | 91–110 | 111–130 | ≥131 |
| Consciousness |  |  |  | Alert |  |  | Confusion, Voice, Pain, Unresponsive |
| Temperature, °C | ≤35.0 |  | 35.1–36.0 | 36.1–38.0 | 38.1–39.0 | ≥39.1 |  |

BP, blood pressure

The chart has a dedicated section (SpO_2_ Scale 2) to be used for patients with hypercapnic respiratory failure (usually due to chronic obstructive pulmonary disease); these patients should have a clinically recommended oxygen saturation of 88–92%.
