# Supplementary material for: Association between Intraoperative Early Warning Score and Mortality and In-Hospital Stay in Lower Gastrointestinal Spontaneous Perforation
Source: Anesthesiol Res Pract. 2023 Aug 29;2023:8910198. doi: 10.1155/2023/8910198 (PMC10480023; doi:10.1155/2023/8910198)
Supplement: Supplementary Materials — S1 Table: National Early Warning Score (NEWS) calculation. S2 Table: Utah Modified Early Warning Score (MEWS) calculation. S3 Table: oxygen saturation (SpO2) to partial pressure of oxygen (PaO2) conversion. S4 Table: vital signs and outcome adjusted for age, sex, preoperative SOFA score, and Charlson comorbidity index. STROBE Statement—Checklist of items that should be included in reports of cohort studies. [file 8910198.f1.zip › S2_Table_file.docx]

S2 Table. Utah Modified Early Warning Score (MEWS) calculation

| Score | 3 | 2 | 1 | 0 | 1 | 2 | 3 |
| --- | --- | --- | --- | --- | --- | --- | --- |
| Temperature, ℃ |  | ≤35.0 | 35.1–35.5 | 35.6–38.0 | 38.1–39.0 | 39.1–39.9 | ≥40.0 |
| Respiration rate, /min | ≤8 |  | 9–11 | 12–20 | 21–25 | 26–29 | ≥30 |
| Pulse, /min | ≤30 | 31–39 |  | 40–100 | 101–110 | 111–130 | ≥131 |
| Systolic BP, mmHg | ≤80 | 81–90 | 91–100 | 101–180 | 181–200 | 201–220 | ≥221 |

BP, blood pressure
