# Supplementary material for: Association between Intraoperative Early Warning Score and Mortality and In-Hospital Stay in Lower Gastrointestinal Spontaneous Perforation
Source: Anesthesiol Res Pract. 2023 Aug 29;2023:8910198. doi: 10.1155/2023/8910198 (PMC10480023; doi:10.1155/2023/8910198)
Supplement: Supplementary Materials — S1 Table: National Early Warning Score (NEWS) calculation. S2 Table: Utah Modified Early Warning Score (MEWS) calculation. S3 Table: oxygen saturation (SpO2) to partial pressure of oxygen (PaO2) conversion. S4 Table: vital signs and outcome adjusted for age, sex, preoperative SOFA score, and Charlson comorbidity index. STROBE Statement—Checklist of items that should be included in reports of cohort studies. [file 8910198.f1.zip › S3_Table_file.docx]

S3 Table. Oxygen saturation (SpO_2_) to partial pressure of oxygen (PaO_2_) conversion

| SpO_2_ (%) | PaO_2_ (Torr) |
| --- | --- |
| 99 | 132 |
| 98 | 104 |
| 97 | 91 |
| 96 | 82 |
| 95 | 76 |
| 94 | 71 |
| 93 | 67 |
| 92 | 64 |
| 91 | 61 |
| 90 | 59 |
| 89 | 57 |
| 88 | 55 |
| 87 | 53 |
| 86 | 51 |
| 85 | 50 |
| 84 | 49 |
| 83 | 47 |
| 82 | 46 |
| 81 | 45 |
| 80 | 44 |
