# Supplementary material for: Association between Intraoperative Early Warning Score and Mortality and In-Hospital Stay in Lower Gastrointestinal Spontaneous Perforation
Source: Anesthesiol Res Pract. 2023 Aug 29;2023:8910198. doi: 10.1155/2023/8910198 (PMC10480023; doi:10.1155/2023/8910198)
Supplement: Supplementary Materials — S1 Table: National Early Warning Score (NEWS) calculation. S2 Table: Utah Modified Early Warning Score (MEWS) calculation. S3 Table: oxygen saturation (SpO2) to partial pressure of oxygen (PaO2) conversion. S4 Table: vital signs and outcome adjusted for age, sex, preoperative SOFA score, and Charlson comorbidity index. STROBE Statement—Checklist of items that should be included in reports of cohort studies. [file 8910198.f1.zip › S4_Table_file.docx]

Additional Table 4. Vital signs and outcome adjusted for age, sex, preoperative SOFA score, and Charlson Comorbidity Index

|  | In-hospital death | Length of hospital stay |
| --- | --- | --- |
|  | Adjusted OR (95% CI), p-value | Adjusted linear regression coefficient (95% CI), p-value |
|  |  |  |
| Start HR | 1.02 (0.99–1.06), p=0.123 | 0.24 (-0.27 to 0.75), p=0.347 |
| End HR | 1.04 (1.01–1.08), p=0.025 | 0.13 (-0.41 to 0.67), p=0.914 |
| Start-to-end delta HR | 1.02 (0.97–1.07), p=0.442 | -0.33 (-1.15 to 0.49), p=0.428 |
|  |  |  |
| Start systolic BP | 1.03 (0.99–1.06), p=0.207 | 0.36 (-0.11 to 0.82), p=0.130 |
| End systolic BP | 1.00 (0.96–1.04), p=0.959 | 0.34 (-0.20 to 0.89), p=0.215 |
| Start-to-end delta systolic BP | 1.01 (0.98–1.05), p=0.362 | 0.10 (-0.35 to 0.55), p=0.661 |
|  |  |  |
| Start mean BP | 1.04 (0.98–1.10), p=0.207 | 0.95 (0.22 to 1.68), p=0.012 |
| End mean BP | 1.00 (0.94–1.07), p=0.940 | 0.75 (0.00 to 1.50), p=0.049 |
| Start-to-end delta mean BP | 1.03 (0.97–1.08), p=0.339 | 0.18 (-0.53 to 0.88), p=0.619 |
|  |  |  |
| Start temperature | 0.90 (0.43–1.86), p=0.768 | -4.34 (-15.15 to 6.48), p=0.427 |
| End temperature | 1.02 (0.26–1.63), p=0.356 | -9.37 (-20.89 to 2.13), p=0.109 |
| Start-to-end delta temperature | 1.35 (0.50–3.67), p=0.552 | 7.88 (-6.68 to 22.44), p=0.285 |
|  | | |

OR, odds ratio; HR, heart rate; BP, blood pressure; 95% CI, 95% confidence interval
